# Long-term evaluation of factors affecting removal torque of microimplants

**DOI:** 10.1186/s40510-021-00383-3

**Published:** 2021-11-15

**Authors:** Ho-Jin Kim, Hyo-Sang Park

**Affiliations:** Department of Orthodontics, School of Dentistry, Kyungpook National University, 2175, Dalgubeoldae-ro, Jung-Gu, Daegu, 41940 Korea

**Keywords:** Microimplant, Removal torque, Long-term stability, Placement method

## Abstract

**Background:**

The current study aimed to evaluate factors affecting the long-term stability of microimplants using removal torque and the correlation between removal torque and clinical variables.

**Materials and methods:**

This research evaluated 703 microimplants placed in 354 patients (mean age: 30.4 ± 12.1 years). The removal torque was evaluated according to various clinical variables including sex, age, placement site, microimplant size, and placement method (self-drilling versus pre-drilling). Pearson correlation and stepwise multiple linear regression analyses were performed to investigate different variables and their association with removal torque.

**Results:**

The mean removal torque was significantly higher in the mandible (4.46 N cm) than in the maxilla (3.73 N cm). The values in the posterior teeth/retromolar areas were significantly higher than those in the anterior teeth area. There were no significant difference in terms of sex. Teenagers had a lower removal torque than older adults in the mandible, but not in the maxilla. Microimplants with a greater length and diameter, except for those with a greater diameter in the maxilla, was associated with a higher removal torque. Regardless of placement torque, the removal torque convergently reached approximately 4 N cm in both placement methods. The removal torque was significantly correlated with screw length in the self-drilling group and with diameter in the pre-drilling group.

**Conclusions:**

Removal torque was related with placement site, age, placement method, and length and diameter of microimplants.

## Background

Orthodontic microimplants have been widely used due to their simple placement and removal, cost-effectiveness, low extensiveness, and absolute nature in anchorage. Accordingly, these devices have become essential in successful orthodontic treatment, particularly for patients with open bite or hyperdivergent growth patterns.

For a successful treatment, the stability of microimplants should be ensured. Previous studies have investigated the factors correlated with the success rate of microimplants [1–7]. Park et al. [1] have reported that mobility, the mandible at the right side, and inflammation were risk factors for the success of microimplants. Some studies have predicted the stability of orthodontic microimplants by evaluating insertion and removal torque [5, 6, 8–18]. To improve primary stability, Motoyoshi et al. [5] recommended a placement torque of 5–10 N cm for orthodontic miniscrews with a diameter of 1.6 mm. In contrast, a previous systematic review did not show a correlation between specific insertion torque levels and better clinical success of orthodontic microimplants [8]. Meanwhile, the long-term stability of miniscrews is associated with removal torque, and several clinical studies have investigated the removal torque of orthodontic miniscrews according to clinical variables, such as age, sex, and placement site and duration [13, 15, 16]. Despite previous studies showing the relation between removal torque and clinical variables, there is minimal information about the effect of microimplant size, various placement sites, and placement method on long-term stability.

The current study aimed to assess factors associated with the long-term stability of microimplants evaluated using removal torque value and to assess correlation between removal torque and clinical variables.

## Materials and methods

**Fig. 1:**
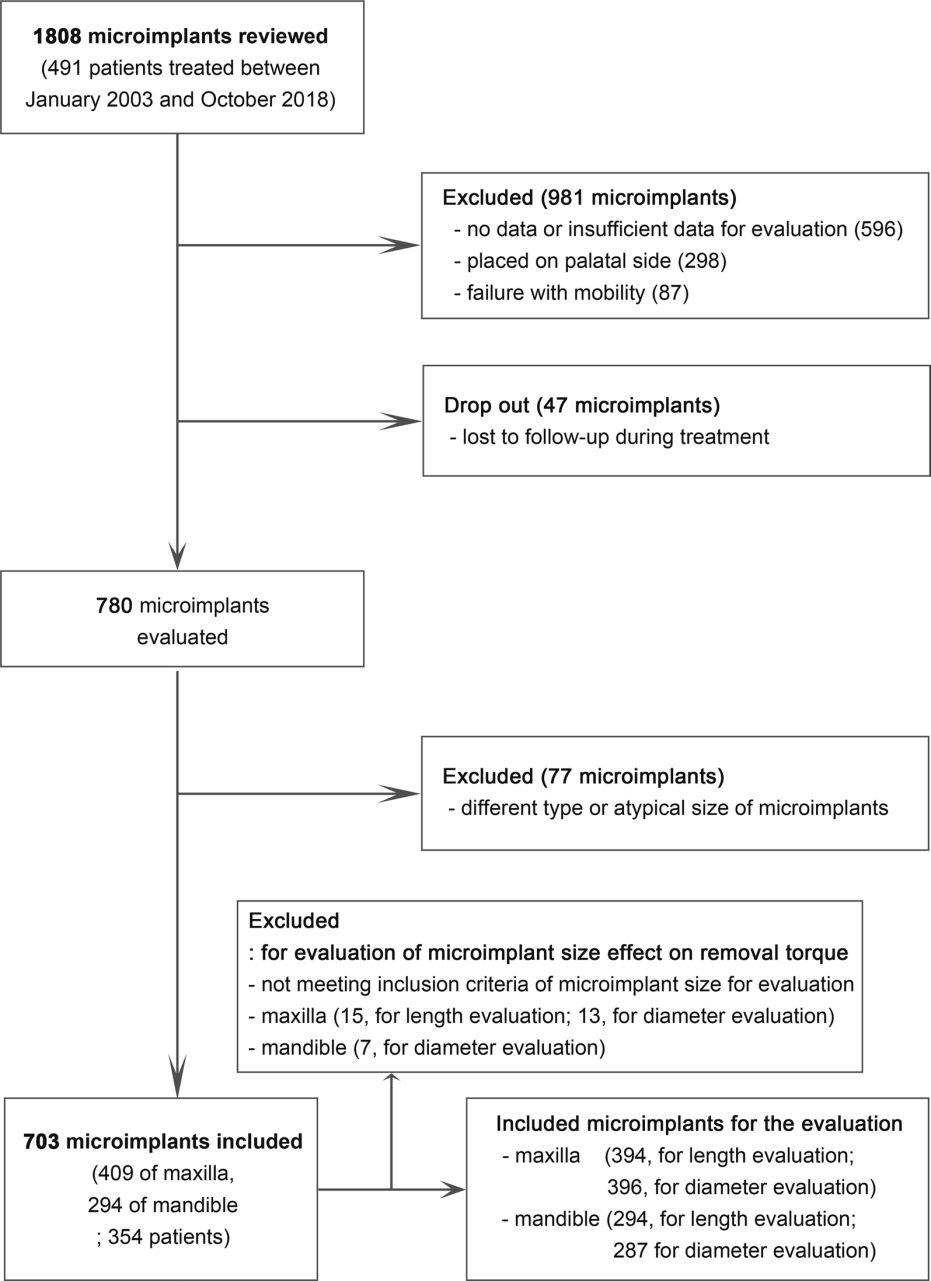
Flowchart of this study

In total, 1808 microimplants placed on 491 patients who visited the Department of Orthodontic, Kyungpook National University Dental Hospital, Daegu, Korea, were reviewed. The participants were treated by one clinician (Park HS) between January 2003 and October 2018. The patients received microimplants as orthodontic anchorage, and they were informed about the risk factors and complications of this procedure. The inclusion criteria were the microimplants used favorably during treatment and having data about removal torque values and relevant variables. Meanwhile, the microimplants without removal torque data, placed on the palatal side, and failed with mobility during treatment were excluded from the study. The process of sampling is shown in Fig. 1 in detail. A total of 981 microimplants that have no or insufficient data for evaluation, were placed on the palatal side, and have failed with mobility were excluded from this study. Another 47 microimplants were dropped out because of missing patients during follow-up or treatment. Regarding the remaining 780 microimplants, 77 microimplants could not be included in this study because of their different type or atypical size. Finally, a total of 703 microimplants placed in 354 patients (117 men, 237 women; mean age: 30.4 ± 12.1 years [range 11.9–75]) were included in this study (Table 1).

**Table 1:** Variables and number of microimplants

Variables		Maxilla (*n*)	Mandible (*n*)
Total	703	409	294
Placement site	Anterior teeth	82	43
	Posterior teeth	327	221
	Retromolar	–	30
Sex	Male	131	92
	Female	278	202
Age	< 20 years	85	46
	20–29 years	205	171
	≥ 30 years	119	77
Length of microimplant	6 mm	21	168
	7 mm	288	126
	8 mm	85	–
Diameter of microimplant	1.3 mm	366	130
	1.4 mm	20	126
	1.5 mm	10	31
Placement method	Self-drilling	352	129
	Pre-drilling	57	165

**Fig. 2:**
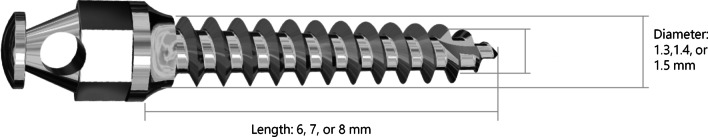
Shape and size of the microimplants used in this study

In addition, to evaluate the effect of size of microimplants on removal torque, those with the following measurements were re-included from the sample of 703 microimplants (Fig. 1): 1.3, 1.4, or 1.5 mm in diameter and 6, 7, or 8 mm in length (Absoanchor, Dentos, Daegu, Korea; Fig. 2). After excluding the microimplants that did not meet the inclusion criteria of size measurements, the numbers of microimplants included for evaluation of length and diameter effects on removal torque were: 394 (for evaluation of length effect) and 396 (for evaluation of diameter effect) microimplants of the maxilla and 294 (for evaluation of length effect) and 287 (for evaluation of diameter effect) microimplants of the mandible.

**Fig. 3:**
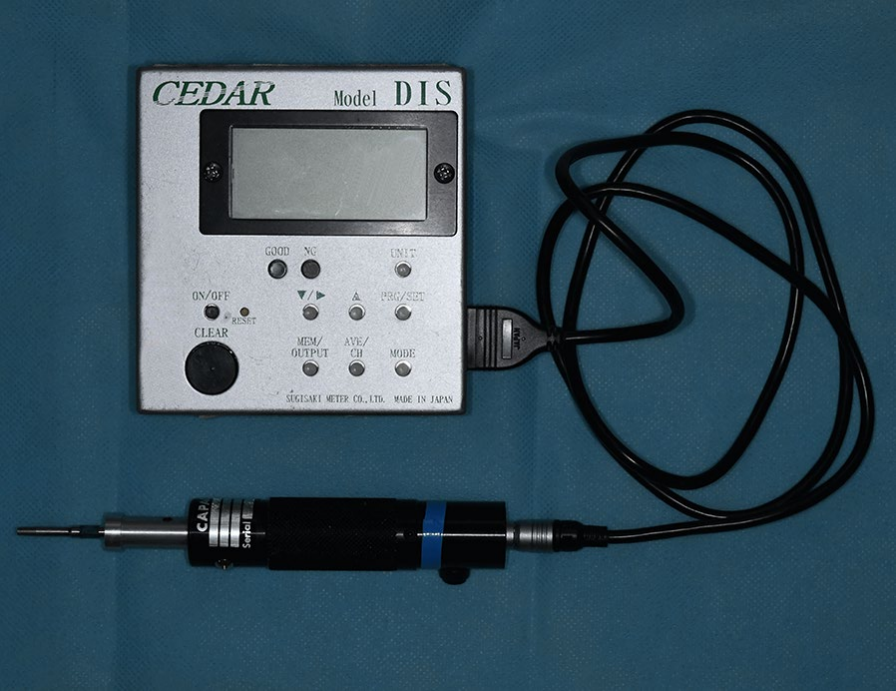
Digital gauge used to measure torque values when placing and removing microimplants

Microimplants were placed into the alveolar bone without an incision or mucoperiosteal flap under local anesthesia. The microimplants were commonly placed at an angle of 30°–50° with the self-drilling or pre-drilling method based on the bone quality of placement site. For the pre-drilling procedure, 0.9- , 1.0- , or 1.1-mm diameter drills were used in the mandible and 0.9-mm drill in the maxilla. All microimplants were placed, and all placement and removal torque values were examined by one author (Park HS). After placement, the microimplants were loaded immediately by light force (approximately 50 g), and the load was increased up to 150–200 gm afterward. The peak removal torque value was measured by the force during first turn in the removal procedure with a digital gauge (DIS-RL05; SUGISAKI METER CO., LTD, Ibaraki, Japan; Fig. 3). The mean duration of microimplant stayed in the bone was 26.39 ± 18.42 months (791.76 ± 552.45 days).

The variables were classified into three groups: host, microimplant, and surgical method factors [19]. Host factors comprised variables such as sex, age, and placement sites. The microimplant factors included length and diameter. The surgical method factor was the placement method, such as self-drilling and pre-drilling. The samples were divided into two groups (the maxilla and mandible). In addition, the placement sites were divided into two in the maxilla (the anterior and posterior areas) and three sites in the mandible (the anterior, posterior, and retromolar areas) depending on the position of microimplants relative to the canines or the mandibular second molars. This study was reviewed and approved by the appropriate institutional review board (IRB no.: KNUDH-2021-03-02-00).

To assess data normality, the Kolmogorov–Smirnov test was performed. If data had a normal distribution, the independent *t*-test or one-way analysis of variance with the post hoc Tukey's test was utilized to compare differences in placement/removal torque between groups. Otherwise, the Kruskal–Wallis test was used. Considering the violated homogeneity of variances across the samples, the Welch’s ANOVA test and Dunnett T3 multiple comparison test were applied. To compare placement and removal torque according to placement torque range and placement method, the paired *t* test was used.

The Pearson correlation was used to evaluate the correlation between the mean removal torque value, age, and length and diameter of microimplants. Subsequently, a stepwise multiple linear regression was utilized to explain the relationship between removal torque, which is a dependent variable, and other factors, which are independent variables. Statistical analyses were performed using the Statistical Package for the Social Sciences software version 22.0 (IBM, Chicago, IL, the USA). A *p* value of < 0.05 was considered statistically significant.

## Results

### Comparison of placement/removal torque values according to various placement sites

**Fig. 4:**
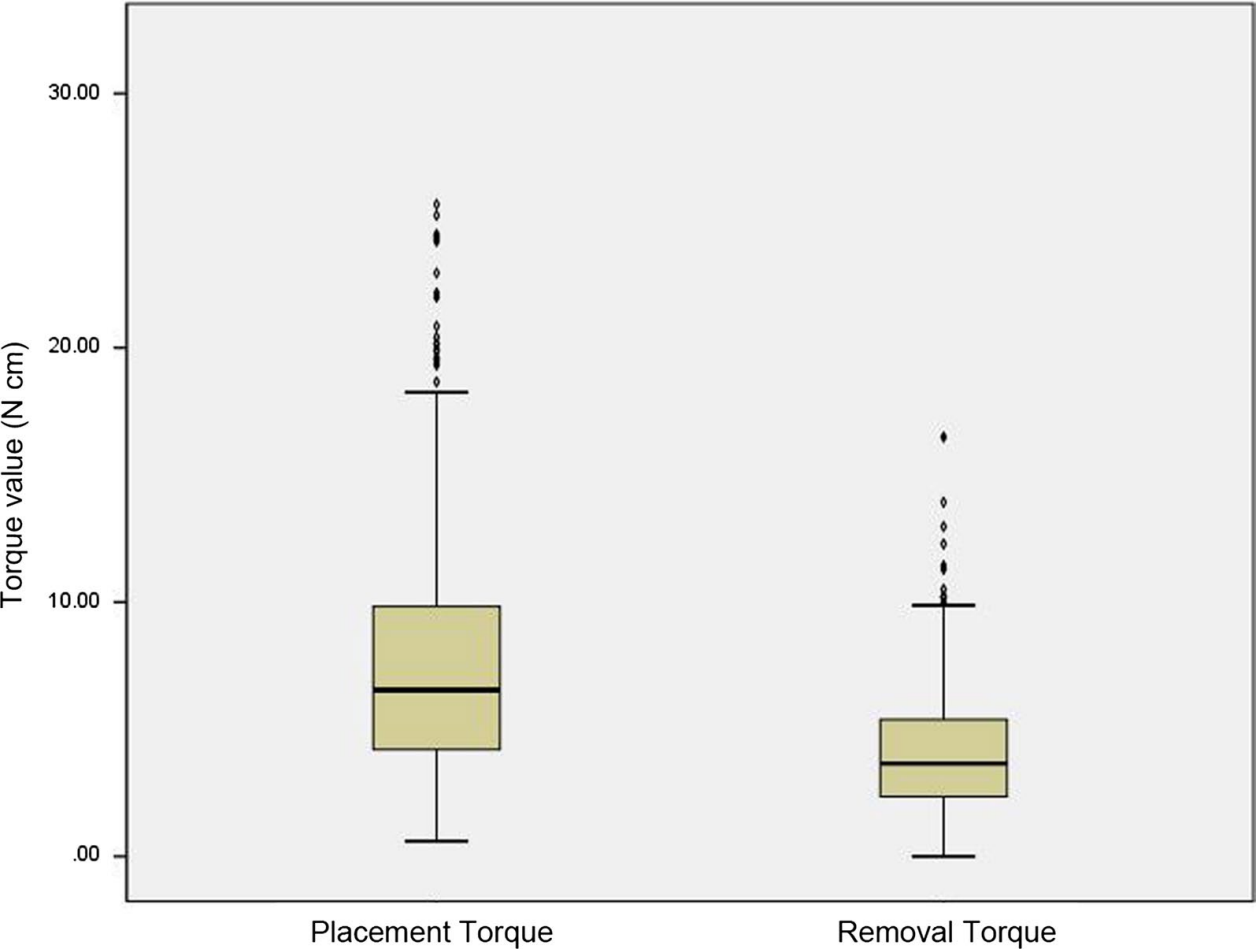
Box plot of the distribution of the total placement and removal torques

**Fig. 5:**
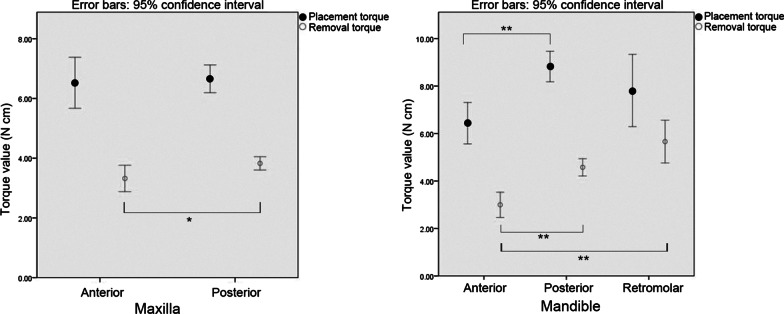
Error bar plot of the mean placement and removal torque values according to placement sites. *p < 0.05, **p < 0.001

The mean placement and removal torque values were 7.36 and 4.03 N cm, respectively (Table 2, Fig. 4). The distribution of removal torque was narrower than that of placement torque. As presented in Table 2 and Fig. 5, the mean placement and removal torque values in the mandible (placement: 8.37 N cm; removal: 4.46 N cm) were significantly higher than those in the maxilla (placement: 6.63 N cm; removal: 3.73 N cm; *p* < 0.001).

**Table 2:**
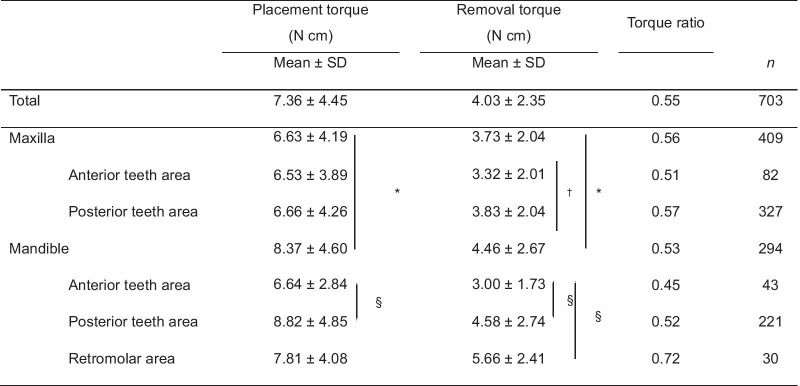
Placement and removal torque values in terms of placement sites

Independent *t*-test was performed for comparison between the maxilla and mandible or the anterior and posterior teeth areas in the maxilla. One-way analysis of variance (ANOVA) with the post hoc Tukey's test was performed for comparison of placement torque between anterior, posterior teeth, and retromolar areas in the mandible. Welch’s ANOVA test and Dunnett T3 multiple comparison test were performed for comparison of removal torque between anterior, posterior teeth, and retromolar areas in the mandible

*Significant difference at *p* < 0.001 between the maxilla and mandible

^†^Significant difference at *p* < 0.05 between the anterior and posterior teeth areas

^§^Significant difference at *p* < 0.001 between the anterior and posterior teeth areas or between the anterior teeth and retromolar areas

In the maxilla, the mean removal torque of the posterior teeth area (3.83 N cm) was higher than that of the anterior teeth area (3.32 N cm; *p* = 0.045).

In the mandible, the placement torque of the posterior teeth area (8.82 N cm) was significantly higher than that of the anterior teeth area (6.64 N cm; *p* < 0.001). The retromolar area (5.66 N cm) had the highest removal torque, followed by the posterior area (4.58 N cm) and the anterior teeth area (3.00 N cm). The removal torque of the mandibular anterior teeth area was significantly lower than that of the posterior teeth or retromolar areas (*p* < 0.001). Meanwhile, the removal torque between the posterior teeth and retromolar areas showed no statistically significant difference (*p* = 0.082).

The torque ratio of removal torque to placement torque was calculated, and the results showed a higher ratio in posterior teeth or retromolar area than in anterior teeth area.

### Comparison of removal torque according to sex and age

As shown in Table 3, there was no significant difference in the mean removal torque value between male and female.

**Table 3:**
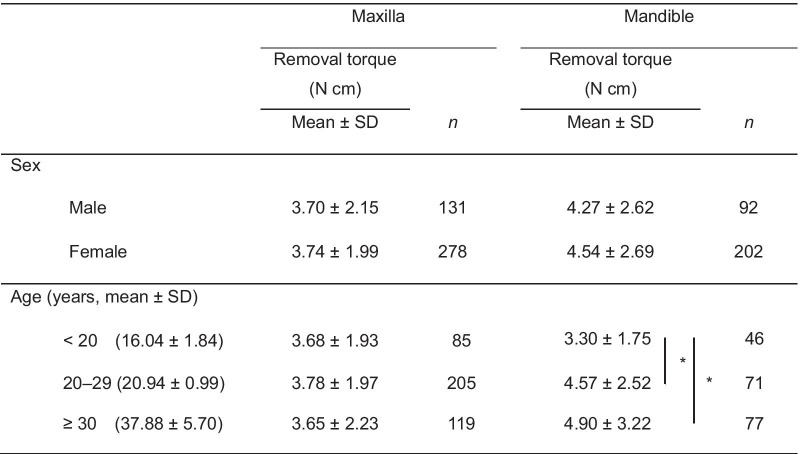
Comparison of removal torque according to sex and age

Independent *t*-test was performed for comparison between men and women

One-way analysis of variance with the post hoc Tukey's test was performed for comparison between age groups

No significant difference between men and women

^*^Significant difference at *p* < 0.05 between different age groups in mandible

The mandibular microimplants of the teenager group (3.30 N cm) had significantly lower removal torque values than the older groups (*p* < 0.05).

### Comparisons of removal torque according to the different lengths and diameters of microimplants

As shown in Table 4, according to the length of microimplants in the maxilla, those with a length of 8 mm (4.30 N cm) had a significantly higher removal torque value than those with a length of 6 mm (3.21 N cm; *p* < 0.05). In the mandible, microimplants with a length of 7 mm (5.57 N cm) had significantly higher removal torque values than those with a length of 6 mm (3.62 N cm; *p* < 0.001).

**Table 4:**
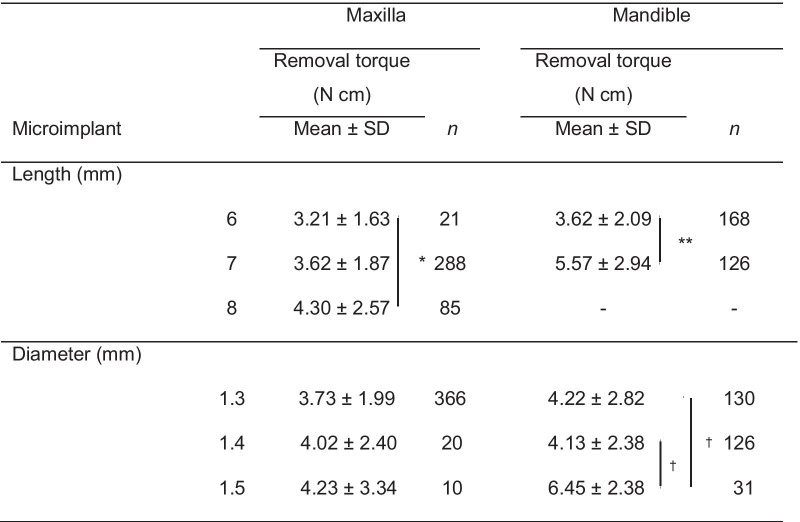
Comparison of removal torque values between microimplants with various lengths and diameters

Welch’s analysis of variance (ANOVA) test and Dunnett T3 multiple comparison test were performed for comparison between groups of microimplant length in the maxilla. Independent *t*-test was performed for comparison between groups of microimplant length in the mandible. Kruskal–Wallis test was performed for comparison between groups of microimplant diameter in the maxilla. One-way ANOVA with the post hoc Tukey's test was performed for comparison between groups of microimplant diameter in the mandible

*Significant difference at *p* < 0.05 between maxillary microimplants with a length of 6 and 8 mm

**Significant difference at *p* < 0.001 between mandibular microimplants with a length of 6 and 7 mm

^†^Significant difference at *p* < 0.001 between mandibular microimplants with a diameter of 1.5 and 1.3 mm or those with a diameter of 1.5 and 1.4 mm

In terms of diameter, there were no significant difference in removal torque values between three diameters in the maxilla. However, in the mandible, the removal torques of microimplants measuring 1.5 mm (6.45 N cm) were significantly higher than those of microimplants measuring 1.3 mm (4.22 N cm; *p* < 0.001) and 1.4 mm (4.13 N cm; *p* < 0.001).

### Comparison between placement and removal torque according to placement torque range and placement method

**Fig. 6:**
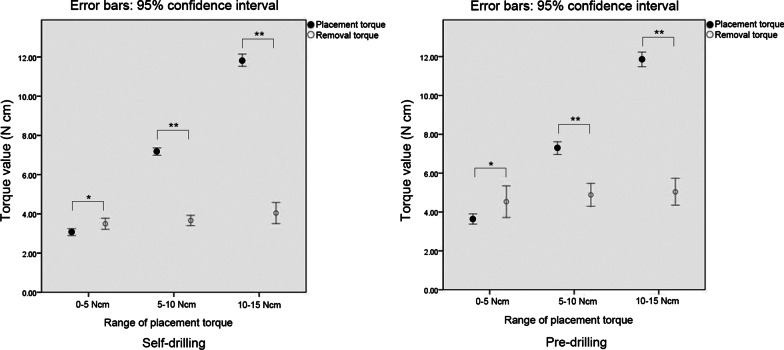
Error bar plot of the mean placement and removal torque values according to the respective ranges of placement torque. *p < 0.05, **p < 0.001

Regardless of placement method, the removal torques significantly differed from the corresponding placement torques in all groups (Table 5, Fig. 6). In the group of microimplants with a placement torque of 0–5 N cm, the removal torque values were significantly higher than the placement torque values (*p* = 0.04). By contrast, in both groups of microimplants with placement torques of 5–10 N and 10–15 N cm, the removal torques were significantly lower than the placement torques (*p* < 0.001).

**Table 5:** Comparison between placement torque and removal torque according to placement torque range and placement method, and comparison of overall torque values between the self-drilling and pre-drilling methods

Placement torque range	Self-drilling (N cm)				Pre-drilling (N cm)			
	Placement torque	Removal torque	Torque ratio	*n*	Placement torque	Removal torque	Torque ratio	*n*
	Mean ± SD	Mean ± SD			Mean ± SD	Mean ± SD		
0–5 N cm	3.07 ± 1.21*	3.49 ± 1.97	1.14	187	3.64 ± 0.96*	4.53 ± 2.98	1.24	54
5–10 N cm	7.17 ± 1.39**	3.66 ± 1.96	0.51	216	7.29 ± 1.47**	4.88 ± 2.65	0.66	79
10–15 N cm	11.83 ± 1.27**	4.04 ± 2.19	0.34	66	11.86 ± 1.44**	5.04 ± 2.63	0.42	58
Total	6.19 ± 3.24^†^	3.64 ± 2.00^†^	0.59	469	7.64 ± 3.43	4.83 ± 2.73	0.63	191

Paired *t*-test was performed for comparison between placement and removal torque. Independent *t*-test was performed for comparison between the self-drilling and pre-drilling methods

*Significant difference at *p* < 0.05 between placement torque and removal torque

**Significant difference at *p* < 0.001 between placement torque and removal torque

^†^Significant difference at *p* < 0.001 between the self-drilling and pre-drilling methods

The mean placement torques had a wider range (from 3.07 to 11.86 N cm) than the mean removal torques (from 3.49 to 5.04 N cm).

The placement torque of 0–5 N cm showed the highest torque ratio followed by that of 5–10 N and 10–15 N cm in both the placement methods.

### Pearson correlation coefficient of removal torque, age, and length and diameter of microimplants according to placement method

There was a significant positive correlations between removal torque and either microimplant length in the self-drilling method (*r* = 0.221, *p* < 0.001) or diameter in the pre-drilling method (*r* = 0.230, *p* < 0.01) (Table 6).

**Table 6:** Correlation coefficients of removal torque, age, and length and diameter of microimplants

	Age	Length of microimplant	Diameter of microimplant
Self-drilling (n = 463)			
Removal torque (mean ± SD: 3.37 ± 2.00 N cm)	0.024	0.221**	0.007
Pre-drilling (n = 220)			
Removal torque (mean ± SD: 4.81 ± 2.82 N cm)	0.004	0.012	0.230*

*p < 0.01, **p < 0.001

### Multiple linear regression analysis of removal torque and relevant variables

A stepwise multiple linear regression analysis was performed to assess removal torque and independent variables such as age and length/diameter of microimplants (Table 7). Results showed that length of microimplants could explain the removal torque in the self-drilling method (adjusted *R*^2^ = 0.047). The analysis could draw the regression equation (*Y*_1_ = −0.925 + 0.663 × *X*_1_; *Y*_1_, predicted removal torque value of the self-drilling microimplant; *X*_1_, length of microimplant).

**Table 7:** Stepwise multiple regression analysis of the effect of associated variables on removal torque values

Variables	Regression coefficient		*p* value
	*B*	*β*	
Self-drilling (*Y*_1_)			
Length of microimplant (*X*_1_)	0.663	0.221	< 0.001
Pre-drilling (*Y*_2_)			
Diameter of microimplant (*X*_2_)	9.065	0.230	0.001

*B* Unstandardized coefficient, *β* standardized coefficient, *R*^2^ coefficient of determination. Adjusted *R*^2^ = 0.047, in the self-drilling group. Adjusted *R*^2^ = 0.048, in the pre-drilling group

Multiple linear regression equation: *Y*_1_ = −0.925 + 0.663 × *X*_1_; *Y*_2_ = −6.582 + 9.065 × *X*_2_

For pre-drilling microimplants, the model showed that the removal torque can be predicted by the diameter (adjusted *R*^2^ = 0.048), and the regression equation was calculated (*Y*_2_ = −6.582 + 9.065 × *X*_2_; *Y*_2_, predicted removal torque value of the pre-drilling microimplant; and *X*_2_, diameter of microimplant).

## Discussion

The primary and long-term stabilities of microimplants are important as they can be used as an anchorage during orthodontic treatment. Removal torque, which is a critical parameter of bone–implant integration, has been evaluated in previous studies about the secondary stability of microimplants [9–18].

To determine factors affecting removal torque, this study investigated 354 patients with 703 microimplants according to size of microimplants, various placement sites, and placement methods. Moreover, the mean placement duration of the microimplants was 26.39 months (791.76 days). Hence, this study period was longer than that of previous studies [13, 16].

Results showed significant differences between placement and removal torques. That is, the mean removal torques (4.0 N cm) were lower than the placement torques (7.4 N cm) (Table 2, Fig. 4). This is consistent with that of a previous study wherein the torque value decreased from 8 to 4 N cm during treatment and a removal torque value of 4 N cm could provide sufficient anchorage [13]. Moreover, it is clinically acceptable based on the recent study. The maxillary anterior and posterior teeth areas and mandibular anterior teeth area had a removal torque value of less than 4 N cm. This may be a cause of concern regarding microimplant failure. However, the maxillary microimplants had a higher success rate, and the mandibular anterior teeth area had greater stability than the posterior teeth area, which is able to contradict the concept of 4 N cm. Other factors including accidental force from occlusion, heavy load, and inflammation can play a role. Hence, removal torque, itself, may not be the only factor affecting microimplant success.

On the other hand, previous studies have reported a removal torque values that are significantly higher than those of the current study. This is likely attributed to the different features of miniscrews, such as diameter, length, and design [15, 16]. The removal torque value was smaller than the insertion torque value mostly in the current study. This finding is in accordance with previous studies [13, 20] regarding torque values and is contrary to another study [15] that demonstrated that the removal torque value was higher than the insertion torque value with a torque ratio > 1.0. This might result from the difference in size, shape (cylindrical or tapered), and surface texture of screws. This too high removal torque value led to four fractured miniscrews during removal caused by partial osseointegration. Notably, excessive osseointegration with a high removal torque is not appropriate for orthodontic microimplants because it may be associated with a higher risk of fracture during removal [21].

The range of removal torque values was relatively narrower than that of placement torque values (Fig. 4). Microimplants placed with low insertion torque showed that the value of removal torque was higher than that of insertion torque, whereas high insertion torque microimplants showed that the removal torque was lower than insertion torque (Table 5). This means that the insertion torques are quite variable according to condition of bones, size of microimplants, and placement method, whereas the range of removal torques is not as wide as that of insertion torques. Once the bone is healed, the contact between the bone and microimplant surface is quite uniform and the removal torque might be proportionate to the surface area of contacts, irregularity of microimplant surface, and bone density.

In keeping with previous studies about placement site, the removal torque of the mandible was significantly higher than that of the maxilla due to the superior bone quality of the mandible [11, 13, 15–17, 22–26]. Hence, a heavier force can be loaded to the microimplants in the mandible if stabilized.

However, studies about the removal torque according to specific placement sites are limited. Thus, in the current study, differences in removal torque according to these sites, such as anterior/posterior teeth area in the maxilla and anterior/posterior/retromolar area in the mandible, were evaluated. The removal torques of the posterior teeth or retromolar areas were significantly higher than those of the anterior teeth area. This is likely attributed to the fact that the thickness and density of the cortical bone in the anterior teeth area are the lowest, and they can increase gradually toward the posterior area (Table 2, Fig. 5) [24–26].

In accordance with previous studies, there were no significant differences in terms of the removal torque of maxillary and mandibular areas between sexes [13, 16]. The age at which the microimplants were removed was significantly relevant to the removal torque in the mandible. The teenagers had lower removal torque values than older adults, presumably due to either lower bone quality or bone immaturity among adolescents, which is similar to an earlier study showing that the cortex of the mandibular alveolus was thicker in adults than in adolescents [27]. However, in the Pearson correlation analysis age did not significantly affect removal torque, as reported previously [13].

Meanwhile, theoretically, a longer microimplant may have a better long-term stability due to a greater osseointegrated interface. The removal torque significantly differed according to the length of microimplants in both jaws in the current study. However, this result should be interpreted with caution because the removal torque was measured from the successfully stayed microimplants. The longer microimplants may have a higher incidence of root contacts, one of the main causes of failure, and may not guarantee higher success rate. In contrast to the result of this research, some previous studies found no significant correlation between removal torque/success rate and implant length [1, 11, 12]. These results may be attributed to the fact that the total length of miniscrews may not exactly coincide with the length inserted into the bone in earlier studies [28]. Moreover, the design of microimplants should be taken into consideration as well. As the length increases, the conical shape used in this study could be rather similar with the cylindrical shape due to a higher length-to-diameter ratio. Generally, the cylindrical shape is well-known for providing better secondary stability based on less bone damage and wider surface area [29–33]. Therefore, this might lead to an increase in the removal torque as well.

Regarding diameter, the experimental study showed that removal torque is proportional to the square of the diameter due to the increased support from the cortical bone [9]. Similarly, in this study, the removal torque values of the mandible significantly differed in terms of diameter. However, in the maxilla, there was no significant difference, which might be attributed to the relatively thin or weak cortical bone of the maxilla [25, 26, 34]. Moreover, the higher risk of root contact with microimplants with a larger diameter during placement or by movement of microimplant during loading might reduce the removal torque in the maxilla. Other possible explanation is the angular placement of microimplants. If microimplants were placed at an angle and with the self-drilling method, this might cause surface bone fracture, which could be aggravated with a larger diameter [35]. Even with increased bone microdamage and root proximity, the bigger microimplants can provide better stability and success rate. However, this may be applicable to the mandible alone. Consequently, microimplants with a larger diameter can be recommended in the mandible, but not in the maxilla. In fact, the success rate of miniscrews with a diameter of < 1.4 mm was higher than that of miniscrews with a diameter of > 1.4 mm in the maxilla [36]. Surface bone damage is remarkably greater in a thicker cortical bone and in a larger diameter in the self-drilling method, and which is more extensive in placement with an angle [28, 35, 37]. Therefore, we recommend the pre-drill method in the angular placement of large microimplants into the mandibular posterior teeth area. Furthermore, notwithstanding the fact that long and wide microimplants have an advantages in terms of stability particularly in the edentulous area, when placing microimplants in the interradicular area, the appropriate length or diameter should be identified.

In a comparison between placement and removal torques, the removal torque had constantly converged into a value of approximately 4 N cm, irrespective of placement torque range and placement method (Fig. 6). Immediately after placing the microimplant, mechanical engagement commonly has an essential role in terms of primary stability. Thereafter, moving to next phase through bone remodeling, overall stability gradually depends on the secondary stability of new bone formation rather than the primary stability of mechanical retention [38]. It seems that the removal torque, bone-to-implant integration, is likely to reach its own inherent value according to determinant factors—host, surgical placement, and implant. Once bone remodeling is done after placement, local bone quality and quantity may have more influence on stability than other factors. The force can be applied to microimplants differently according to sites which have different bone density and thickness. Therefore, the force should be lower in the anterior teeth and the maxilla than in the posterior teeth area and the mandible.

To validate factors affecting removal torque, a Pearson correlation analysis of clinical variables was performed. Interestingly, the removal torque was positively correlated with the length of microimplant in the self-drilling group and diameter in the pre-drilling group. At the placement site requiring pre-drilling procedure, there is a thick and hard cortical bone that may affect stress distribution. On the contrary, when placing using the no drill method at the site of weak cortical bone, the cancellous bone may have more influence on stability. Therefore, microimplant diameter could significantly affect stability in the pre-drilling group as did length in the self-drilling group. Moreover, bone damage can occur during the placement of microimplants at an angle to the bone surface [35]. Because the microimplants were mostly placed at an angle of 30°–50° to the bone surface in this study, surface bone damage might be more extensive using the self-drilling method, and this might reduce bone contact areas and affect removal torque negatively.

Park et al. [26] have emphasized that the length of screws was highly decisive to those stability in the maxilla, and the diameter of screws in the mandible. This finding was in accordance with that of this study because the placement method (self-drilling and pre-drilling) was determined depending on bone quality of the site. That is, self-drilling and pre-drilling methods were preferred in the maxilla and mandible, respectively.

We observed that the duration of microimplant stayed in the bone showed a positive significant correlation with removal torque in the self-drilling group (*r* = 0.249; *p* < 0.001) (unpublished data), but not in the pre-drilling group. It is assumed that bone microdamage during placement and subsequent bone repair were significantly higher in the self-drilling group rather than in the pre-drilling group. This may positively influence the increase of the removal torque with time in the self-drilling group. An earlier study also demonstrated that the removal torque was not correlated with the placement period in pre-drilling orthodontic miniscrews [13].

In a multiple regression analysis, length and diameter were the variables associated with removal torque in the respective placement method groups. However, this may not be applicable to larger and longer miniscrews due to root contacts when placed into the interradicular space.

Although this study has found a significant relationship between removal torque and relevant variables, it has a limitation that should be considered. When placing screws, the protocol for selecting particular types, such as length and diameter, according to placement sites—considering its anatomy and bone quality—might have affected the results. Moreover, because the effects of length and diameter of microimplants could be interacted and influence the torque values, their effects should be interpreted with caution, although profound statistical analyses were done.

In future studies, it should be performed to compare the removal torque of screws in use during treatment and those in less use during retention period.

## Conclusions

The removal torque values of microimplants in the posterior teeth or retromolar areas were significantly higher than those of in the anterior teeth area. The older groups showed significantly higher removal torque values than the teenager group in the mandible. Both long and wide microimplants had significantly high removal torque values, except for the wide ones in the maxilla. The constant value of removal torque (approximately 4 N cm) was observed irrespective of placement torque and placement method. The length and diameter of microimplants were correlated with removal torque in the self-drilling and pre-drilling groups, respectively.

## Data Availability

Not applicable.
